# Analysis of the Magnetic Field Magnetoinductive Wave Characteristics of a Wireless Power Transfer System

**DOI:** 10.3390/s22249839

**Published:** 2022-12-14

**Authors:** Jianwei Kang, Deyu Zeng, Jie Lu, Xiangyang Shi

**Affiliations:** Key Laboratory of Testing Technology for Manufacturing Process, Ministry of Education, School of Manufacturing Science and Engineering, Southwest University of Science and Technology, Mianyang 621010, China

**Keywords:** wireless power transfer, magnetic field, wave characteristics, magnetoinductive wave

## Abstract

This study analyzes the magnetic field wave characteristics of a wireless power transfer (WPT) system from a time-varying view in the nonradiative near field. Phenomena of both forward and backward traveling waves were found. These wave phenomena refer to magnetoinductive waves (MIWs) according to the findings in this study and MIW theory and characteristics. A traditional MIW only appears in the MIW waveguide, which is always constructed by many parallel coils. However, this study analyzed MIWs in a two-coil WPT system, proving that MIWs exist not only in a multi-coil system but also in a basic two-coil system. The velocity of MIWs, a kind of a phase velocity, was calculated. An approximate equation for evaluating wave velocity is proposed. Furthermore, the MIWs in the two-coil WPT system were extended into a more general situation. In this general situation, two separated standing waves were set, and a traveling wave was generated by those two standing waves. The result explains the mechanisms of MIWs in a general situation from a time-varying view. Lastly, a simulation was conducted to verify the accuracy of the study. The results demonstrated that MIWs exist, and the approximate equation is correct. This study presents a novel view on the mechanisms of the WPT system from a wave view.

## 1. Introduction

Wireless power transfer (WPT) systems are prospective power transfer technologies in electrical areas [1,2,3,4]. The analysis and calculation of the electromagnetic field of WPT systems has received considerable attention from researchers. Previous research includes analyses on magnetic field calculation methods [5,6,7], calculations of magnetic fields to optimize the system parameters [8], electromagnetic field evaluation [9], and coils and shielding design [10,11,12,13]. 

The characteristics of the magnetic field of the coil system are a hot topic [14,15], especially in the WPT system. The magnetic field, via magnetic field flux lines, was analyzed and the field around a WPT system was defined as a stray magnetic field in [16]. The stray field at one point represents either the leakage field or the coupling field. The stray or leakage magnetic field was analyzed in [17,18] to cancel the stray or leakage magnetic field via different additional coils. The direction of the magnetic field has also been analyzed in [19]. These studies analyzed the magnetic field of a WPT system in the condition of steady state. They present an enhanced understanding of the characteristics of the magnetic field in the WPT system, but time-varying characteristics should also be considered to investigate the magnetic field characteristics more comprehensively.

Time-varying characteristics are important characteristics of the magnetic field in a WPT system. The distributions of magnetic fields at different time degrees are presented in [20], which is one of the early studies on time-varying characteristics. The findings of polarization characteristics [21] and non-sine wave characteristics [22] present a deep understanding of the instantaneous characteristics of the magnetic field in the WPT system. These analyses were all from the field view under a frequency from several kHz to MHz, and the geometric size of the WPT system was much smaller than the wavelength of the electromagnetic field used. This condition means that the magnetic field was in the nonradiative near field, and the analyses are from a field view and not from a wave view.

Furthermore, researchers also analyzed the magnetic field from the wave view and mainly discussed the radiation characteristics of the electromagnetic field in a WPT system. The Fresnel zone was introduced to depict the electromagnetic field around a WPT system in the radiative near field [23]. Gaussian and other beams were used to establish a WPT system in the Fresnel zone to obtain enhanced efficiency under 10 GHz [24]. Research in [25] analyzed a radiative WPT system at a 1.9 GHz frequency.

It is generally agreed that if the wavelength of a magnetic wave is much larger than the geometric size of a WPT system, then no wave or radiative characteristics exist [26]. Hence, the magnetic field analysis in the nonradiative near field is mainly in the field view not the wave view. The magnetic field analysis in the radiative near field is under wave view. However, even in a nonradiative near field condition, the wave view can be used to analyze a WPT system, and this presents further insight into the time-varying characteristics of the magnetic field in the WPT system. This idea is a result of the WPT system being a coupling system in the near field. Under these conditions, a magnetoinductive wave (MIW) will appear.

An MIW is generated from the MIW waveguide, and the geometric size of the structure is smaller than the free space wavelength [27]. A 1D MIW waveguide is constructed using several parallel coils, and an MIW travels in those coils. The MIW exhibits both forward and backward waves [27]. A WPT system that contains relay coils is also regarded as an MIW waveguide [28,29]. The analysis of MIWs, up to now, has mainly been on the impedance of the system [30] and the application of MIWs [31].

However, these analyses are based on WPT systems that contain relay coils. Whether MIW exists in a basic WPT system that contains only two coils, and what the forward and backward waves of the MIW are in the WPT system, are unclear. Moreover, other properties of the MIW in the WPT system have not been analyzed yet. What the relationship between the time-varying characteristics and MIWs is, and how to quantify MIWs are problems that need to be solved.

In this study, our goal is to analyze the magnetic field characteristics from an MIW perspective. The MIW in a two-coil WPT system was analyzed from a time-varying perspective. The forward and backward waves of the MIWs were found and visualized. A wave traveling velocity was calculated to quantify the MIWs, and an approximate equation is proposed to calculate the wave velocity. This velocity was not the electromagnetic field traveling velocity in free space, but rather a phase velocity. Moreover, the MIW in the WPT system was extended to a more general situation. The result was that the two standing waves generated a traveling wave. This result presented a mechanism of the generation of MIWs from a general situation. This study presents a novel view on the characteristics of the magnetic field in the WPT system, which can be used to enhance the understanding of the mechanisms of the WPT system. This study also expands on the current knowledge of MIWs.

The remainder of this paper is arranged as follows: In Section 2, the magnetic field intensity is deduced and the wave phenomenon is presented. In Section 3, the components of the magnetic field are analyzed, and MIWs are confirmed and quantified by velocity. An approximate equation of velocity is also proposed. Section 4 presents a more general situation to illustrate the mechanism of MIWs. In Section 5, the theoretical analysis is proven using the ANSYS Electromagnetics Suite. Lastly, Section 6 presents the conclusion.

## 2. Wave Phenomenon in WPT

### 2.1. Magnetic Field Intensity of the WPT System

**Figure 1:**
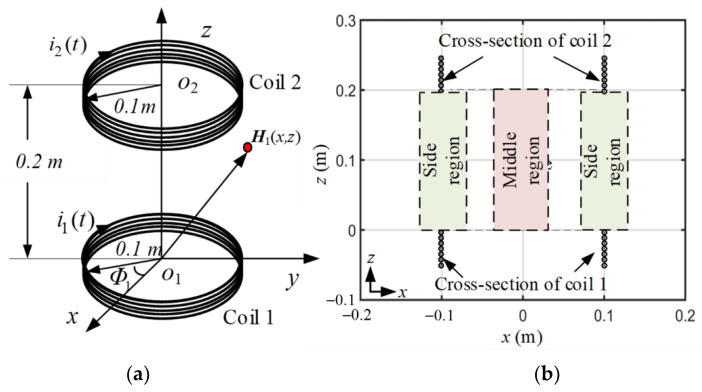
Two-coil WPT system. (**a**) the position of the two-coil WPT system. (**b**) the cross-section on the xoz plane of the WPT system.

A two-coil eight-turn WPT system is shown in Figure 1. The fields on the xoz plane due to the axial symmetry of the system will be calculated and analyzed. Coils 1 and 2 represent the transmitter and receiver coils, respectively. The coil radius is 0.1 m, and the distance of the two coils is 0.2 m. In Figure 1b, two regions are plotted for further analysis. One region is called the middle region, and the other is the side region. The MIW characteristics in those regions will be discussed.

The currents in the two coils are as follows:

(1) $$i_{1}(t)\;=\;I_{1}\cos(\omega t)$$

(2) $$i_{2}(t)\;=\;I_{2}\cos(\omega t\;+\;∆\phi )$$

where ω is the angular frequency, and the frequency of the WPT system is 1 MHz. $I_{1}$ and $I_{2}$ are the magnitudes of the two currents, which are set to 1 A. ∆ϕ is the current phase difference. The power transferring condition is ∆ϕ ≠ 0 according to the theory of the WPT system. The $I_{1}$ and $I_{2}$ can be measured according to the experiment, and they are treated as two independent current sources in coil 1 and 2, respectively. According to the superposition law of the electromagnetic field, the total magnetic field can be superposed by two independent fields generated by the two currents.

The magnetic field intensities generated by coils 1 and 2 are $H_{1}(x,z,t)$ and $H_{2}(x,z,t)$, respectively. In the analysis model, both currents in coils 1 and 2 are the sources of the magnetic field. $H_{1}(x,z,t)$ is superposed by the x-component $H_{x1}(x,z,t)$ and the z-component $H_{z1}(x,z,t)$. That is,

(3) $$H_{1}(x,z,t)\;=\;H_{1x}(x,z,t)e_{x}\;+\;H_{1z}(x,z,t)e_{z}$$

where $e_{x}$ and $e_{z}$ are the unit vectors of the x and z directions, respectively, and

(4) $$H_{x1}(x,z,t)\;=\;I_{1}\cos(\omega t)T_{x1}(x,z)$$

(5) $$H_{z1}(x,z,t)\;=\;I_{1}\cos(\omega t)T_{z1}(x,z)$$

where $T_{x1}(x,z)$ and $T_{z1}(x,z)$ are the geometrical functions, which are defined in [22] as follows:

(6) $$T_{x1}(x,z)\;=\;\frac{1}{4\pi }\int_{0}^{2\pi }\frac{\cos\Phi_{1}\cdot R(z\;-\;z_{1})d\Phi_{1}}{{\left[x^{2}\;+\;R^{2}\;-\;2xR\cos\Phi_{1}\;+\;{\left(z\;-\;z_{1}\right)}^{2}\right]}^{3/2}}$$

(7) $$T_{z1}(x,z)\;=\;\frac{1}{4\pi }\int_{0}^{2\pi }\frac{(R^{2}\;-\;\cos\Phi_{1}Rx)d\Phi_{1}}{{\left[x^{2}\;+\;R^{2}\;-\;2xR\cos\Phi_{1}\;+\;{\left(z\;-\;z_{1}\right)}^{2}\right]}^{3/2}}$$

where (x,z) is the field point, and $\Phi_{1}$ is the source point degree of coil 1 shown in Figure 1a. This degree is divided 2π into several equal integral parts, and each integral part is equal to $\Phi_{1}$. $z_{1}$ is the source z-position of coil 1.

The magnetic field intensity generated by coil 2 is as follows:

(8) $$H_{2}(x,z,t)\;=\;H_{x2}(x,z,t)e_{x}\;+\;H_{z2}(x,z,t)e_{z}$$

and

(9) $$H_{x2}(x,z,t)\;=\;I_{2}\cos(\omega t\;+\;∆\phi )T_{x2}(x,z)$$

(10) $$H_{z2}(x,z,t)\;=\;I_{2}\cos(\omega t\;+\;∆\phi )T_{z2}(x,z)$$

where $T_{x2}(x,z)$ and $T_{z2}(x,z)$ are the geometrical functions of coil 2 [22]. They are as follows:

(11) $$T_{x2}(x,z)\;=\;\frac{1}{4\pi }\int_{0}^{2\pi }\frac{\cos\Phi_{2}\cdot R(z\;-\;z_{2})d\Phi_{2}}{{\left[x^{2}\;+\;R^{2}\;-\;2xR\cos\Phi_{2}\;+\;{\left(z\;-\;z_{2}\right)}^{2}\right]}^{3/2}}$$

(12) $$T_{z2}(x,z)\;=\;\frac{1}{4\pi }\int_{0}^{2\pi }\frac{(R^{2}\;-\;\cos\Phi_{2}Rx)d\Phi_{2}}{{\left[x^{2}\;+\;R^{2}\;-\;2xR\cos\Phi_{2}\;+\;{\left(z\;-\;z_{2}\right)}^{2}\right]}^{3/2}}$$

where (x,z) is the field point, and $\Phi_{2}$ is the source point degree of coil 2. $z_{2}$ is the source z-position of coil 2.

The superposed magnetic field of coils 1 and 2 is obtained as follows:

(13) $$\begin{array}{ll} H(x,z,t) & \;=\;H_{1}(x,z,t)\;+\;H_{2}(x,z,t)\\ & \;=\;H_{x}(x,z,t)e_{x}\;+\;H_{z}(x,z,t)e_{z} \end{array}$$

where $H_{x}(x,z,t)$ and $H_{z}(x,z,t)$ are the x and z components of the superposed magnetic field, respectively, and can also be expressed as follows:

(14) $$H_{x}(x,z,t)\;=\;H_{x1}(x,z,t)\;+\;H_{x2}(x,z,t)$$

(15) $$H_{z}(x,z,t)\;=\;H_{z1}(x,z,t)\;+\;H_{z2}(x,z,t)$$

### 2.2. Wave Phenomenon in the WPT System

The magnitude of H(x,z,t) is calculated as follows:

(16) $$H_{mag}(x,z,t)\;=\;\sqrt{{H_{z}}^{2}(x,z,t)\;+\;{H_{z}}^{2}(x,z,t)}$$

**Figure 2:**
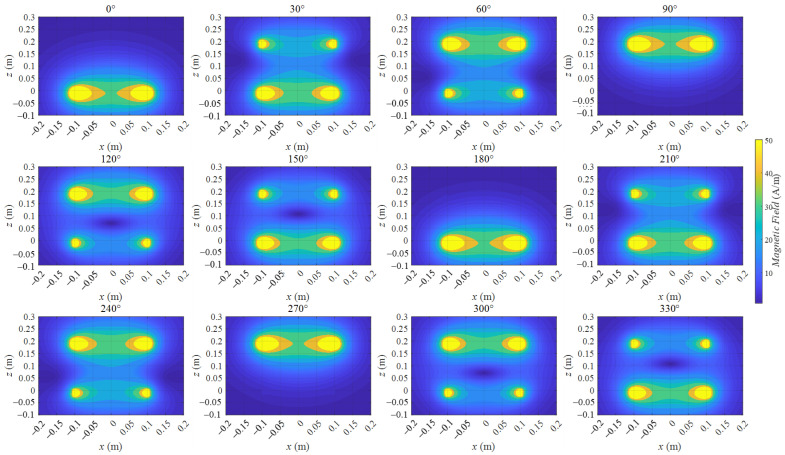
Magnetic field distribution on the xoz plane varying with time phases from 0° to 330° in 30° increments. Figure 2 presents a whole cycle period from 0° to 330°. The degree symbol stands for the time in one period.

The magnetic field is calculated using (16) and shown in Figure 2. The working currents of coils 1 and 2 are both set to 1 A. The current phase difference of current 1 and current 2 is set to −90°.

Figure 2 presents a whole cycle period from 0° to 330°. The degree symbol stands for the time in one period. The different degrees indicate different times in one period of the current. Hence, the distributions of the magnetic field indicate the instantaneous distribution at different times.

Initially, the magnetic field period was half of the current period. Hence, the distributions from 0° to 180° have the same variation from 180° to 360°. In the remainder of this paper, only 0° to 180° is considered.

A wave phenomenon occurred in the instantaneous magnetic field distributions. In the distribution from 90° to 180°, a dark bule area moved from coil 1 to coil 2 along the middle line x = 0 m of the middle region shown in Figure 1b. The dark blue area stands for the low value of the magnetic field, and it can also be regarded as a trough of some kinds of waves. When the time changed, this trough moved from coil 1 to coil 2. This phenomenon is similar to the movement of an equiphase plane in a plane wave situation, but this wave phenomenon is more complicated than that in a plane wave situation.

If a wave characteristic exists, questions relating to what the wave is, how the phenomenon occurs, and how to quantify the wave characteristics arise. The following sections answer these questions.

## 3. Analysis of the Wave Characteristic

### 3.1. Z-component Wave Characteristic

**Figure 3:**
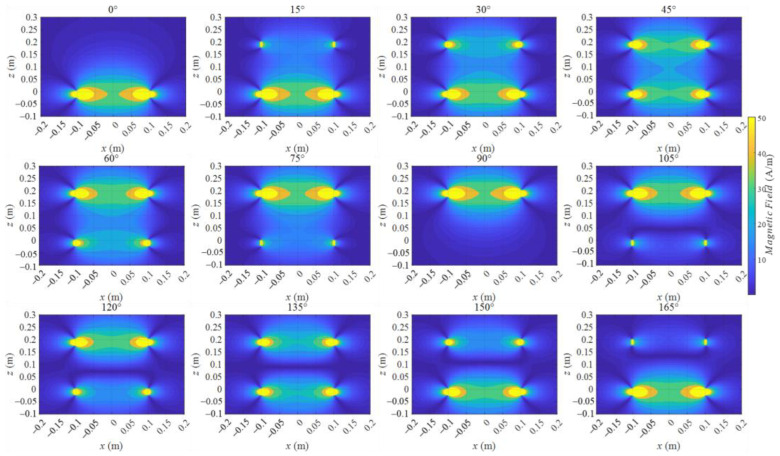
Component distributions varying from 0° to 180° in 15° intervals.

In the middle region, which was near the line of x = 0 and on the line of x = 0 m shown in Figure 1b, $H_{x}(x,z,t)$ was smaller than $H_{z}(x,z,t)$. Hence, $H_{x}(x,z,t)$ dominates $H_{mag}(x,z,t)$. This result shows that if $H_{mag}(x,z,t)$ possess a wave phenomenon in the middle region, $H_{z}(x,z,t)$ must exhibit the same wave phenomenon. Therefore, $H_{z}(x,z,t)$ distributions at different times from 0° to 180° were calculated and are shown in Figure 3. The dark blue area is parallel to the *x*-axis that moves from coil 1 to coil 2 when the time is from 90° to 180°, implying that the wave phenomenon exists.

**Figure 4:**
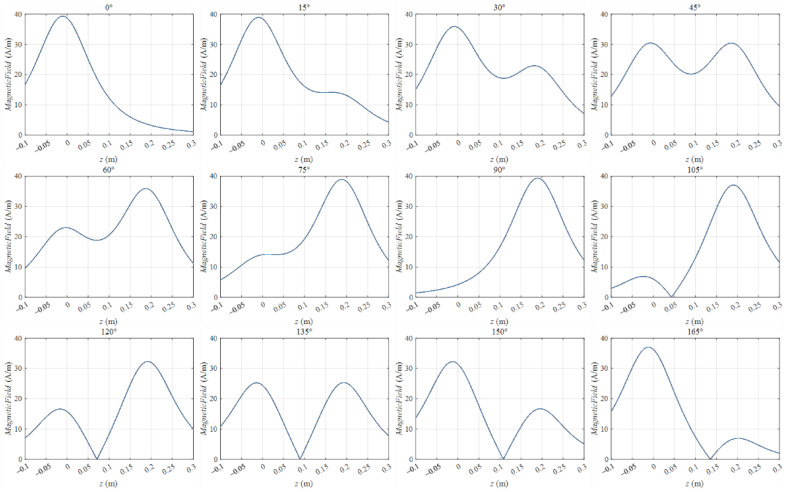
On line x = 0 m from z = −0.1 m to z = 0.3 m varying from 0° to 180°.

The magnetic field $H_{z}(x,z,t)$, on line x = 0 from z = −0.1 m to z= 0.3 m, is shown in Figure 4 to present a clearer view of the instantaneous variation characteristics to analyze the wave phenomenon. We call this line the middle line for convenience, and this line is shown in Figure 1. 

From 105° to 165°, a zero point emerged on each curve of the different time phases. The zero point emerged in the whole half period, except at the degree equal to 90° or 180°. The z coordinate of the zero point varied from z = 0.05 m to z = 0.14 m when the time degree varied from 105° to 165°. This phenomenon was the wave motion, and the moving direction was from coil 1 to coil 2, which was the power transfer direction.

Additionally, a trough emerged from 15° to 75°. In fact, this trough emerged in the entire half period, except at the degree equal to 0° or 90°. The trough exhibited a wave motion characteristic similar to that of the zero point. It moved from z = 0.12 m to z = 0.05 m when the time degree varied from 15° to 75°. However, this moving direction was opposite to the moving direction of the zero point. Moreover, this trough wave motion phenomenon was difficult to find in the distribution figures of $H_{z}(x,z,t)$ because the trough region was submerged by the near area whose value was near the trough value. Only in the line variation situation can one find the trough moving phenomenon, but the trough moving phenomenon also existed in the WPT space.

This result showed that in a period of the magnetic field, two wave motions occurred. One moved from coil 2 to coil 1 in the first half period, and the other moved from coil 1 to coil 2 in the last half period. Those two waves were the backward and forward waves of MIW according to the characteristics of MIWs [27]. The wave phenomena shown in Figure 4 prove the existence of the MIW in the two-coil system.

### 3.2. Quantification of the Z Component in MIW Characteristics

Wave motion velocity is a suitable quantity to quantify moving characteristics, including MIWs, and it was used to quantify the wave motion characteristics of the magnetic field in the WPT system in this study. A similar displacement–time curve was initially obtained, then, the velocity–time curve was calculated.

The minimum value point of $H_{z}(x,z,t)$ curve in each time degree step was initially selected. The time degree step was set to 5°. The minimum value points stand for the trough point or the zero point on the middle line from z = 0 m to z = 0.2 m, given that the wave motion phenomenon mainly emerged in that region, as shown in Figure 4. The position from z = 0 m to z = 0.2 m was in the region between the two coils.

**Figure 5:**
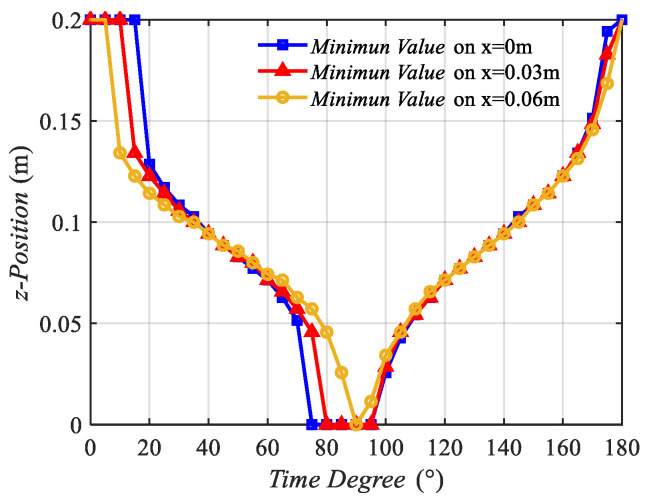
Z position of the minimum value points of $H_{z}(x,z,t)$ on three middle lines varying with time degree.

Then, the z position of the minimum value point was recorded, and the z position and time degree curve, which was similar to the displacement–time curve, is presented in Figure 5.

Two additional lines near the middle line were selected to illustrate that the wave motion appeared not only on the middle line but also in that middle region. The two lines are the line on x = 0.03 m and the line on x = 0.06 m. $H_{z}(x,z,t)$ on those lines varied from the time degree of 0° to 180°.

Figure 5 contains three curves, which are in the same tendency. From 0° to 90°, the z positions of the three curves decreased from 0.2 m to 0 m, indicating that the minimum value point moves from coil 2 to coil 1. In this process, the minimum value point stands for the trough plotted in Figure 4. However, from 90° to 180°, the curves increased from 0 m to 0.2 m, indicating that the minimum value point standing for the zero point shown in Figure 4 moved from coil 1 to coil 2.

**Figure 6:**
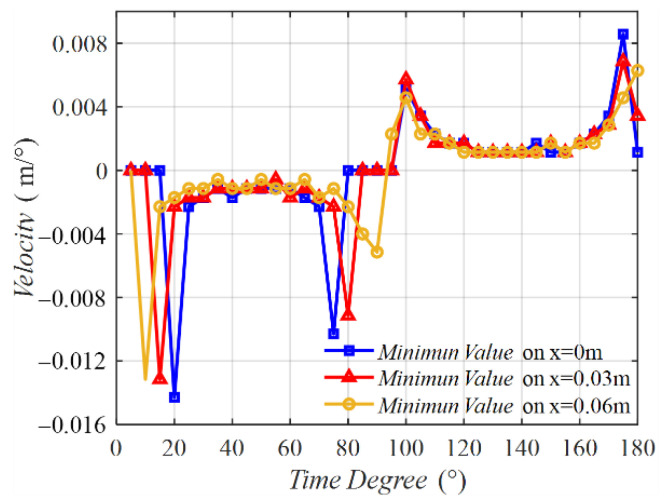
Velocity on three middle lines varying with time degrees.

**Figure 7:**
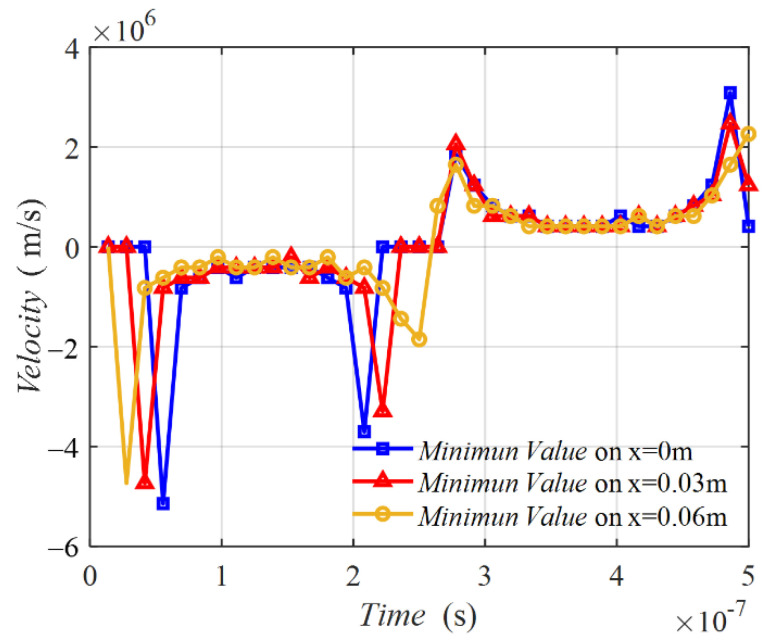
Velocity on three middle lines varying with time.

Figure 6 shows the velocities of the minimum points on the three middle lines varying with time degrees. The three velocity curves possessed a similar tendency. The curve was not a line parallel with the horizontal line, implying that the velocity was not a constant. In the time of 0° to 90°, the velocity was negative, meaning that the minimum value point was moving from coil 2 to coil 1. At the initial time near 0° and the final time near 90°, two peaks emerged. From 90° to 180°, the velocity was positive, indicating that the minimum value point was moving from coil 1 to coil 2. Two peaks also exist in this half period. Figure 7 shows the MIW velocity with time, which is transferred from the results in Figure 6 of time degrees to time. This result shows the velocity in m/s, which presents a better understanding of the MIW velocity.

The average velocities of different lines and in two periods were calculated and are shown in Table 1. The average velocity of all the three lines in the first half period was equal to $8.31\;\times \;10^{5}\;m/s$. The average velocity in the last half period was $1.03\;\times \;10^{6}\;m/s$, which was larger than that in the first half period. The total average velocity $\bar{v}$ was $9.28\;\times \;10^{5}\;m/s$.

This average velocity of the minimum value point was the average velocity of the wave motion characteristics and was the velocity of MIWs in the WPT system. This velocity was considerably less than the wave velocity of the plane wave in space, which was $3\;\times \;10^{8}\;m/s$. This result indicates that this wave motion velocity of the MIW in the WPT system is a kind of phase velocity.

Additionally, we found an equation that can be used to calculate the average velocity of the magnetic field in the WPT system. The average velocity of the wave motion $\bar{v}$ could be approximately calculated using the following equation:

(17) $$\bar{v}\;=\;4df$$

where d is the distance between coils, and f is the system frequency. The result of (17) in the situation of this paper is $\bar{v}\;=\;4\;\times \;0.2\;\times \;10^{6}\;m/s\;=\;8\;\times \;10^{5}\;m/s$, which was close to the $9.28\;\times \;10^{5}\;m/s$ calculated using theoretical analysis. Equation (17) can be used to calculate the wave motion of the magnetic field in a WPT system.

### 3.3. X Component Wave Characteristic

We have found that MIW appears in the middle region of the WPT system space. This wave motion appeared in the whole magnetic field period. However, the above MIW belonged to $H_{z}(x,z,t)$. Whether the MIW also exists in $H_{x}(x,z,t)$ is unknown. We also analyzed the wave phenomenon of $H_{x}(x,z,t)$, and the MIW also exists.

In the side regions (the side region is shown in Figure 1) of 30° and 60° subfigures of Figure 2, two dark blue areas appear, and they move from coil 2 to coil 1 when the time degree increases from 0° to 90°. This dark blue motion in the side regions was similar to that in the middle region, but the direction was opposite. Although this dark blue area motion belonged to $H_{mag}(x,z,t)$, $H_{x}(x,z,t)$ dominated $H_{mag}(x,z,t)$ in those side regions. This result means that $H_{z}(x,z,t)$ was very small in those side regions, as illustrated in Figure 3.

**Figure 8:**
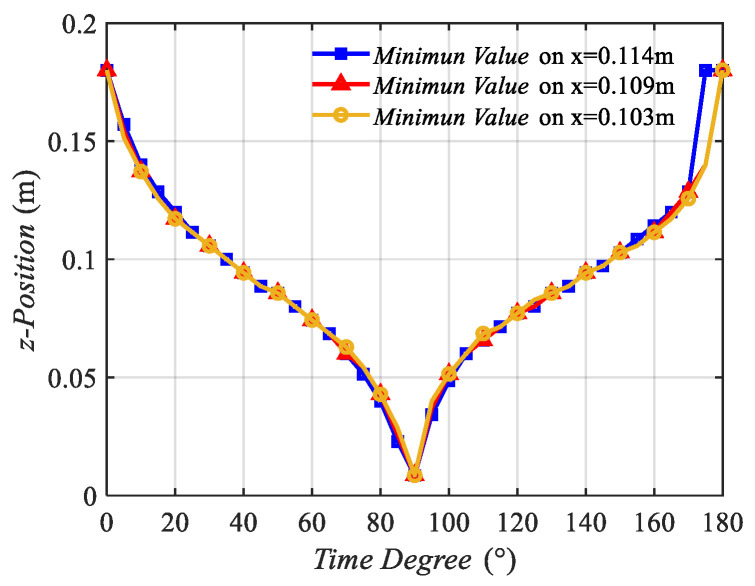
Z position of the minimum value points of $H_{x}(x,z,t)$ on three side lines varying with time degrees.

**Figure 9:**
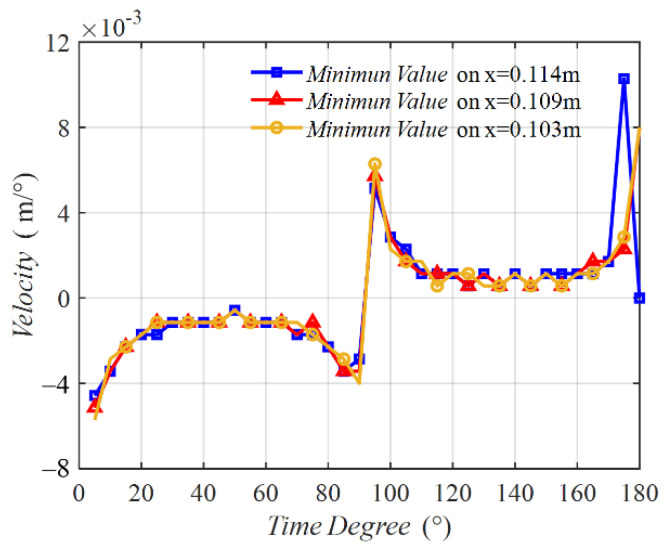
Velocity on three side lines varying with time degrees.

Three side lines, which are x = 0.114 m, x = 0.109 m, and x = 0.103 m, on the xoz plane were selected to analyze the MIW characteristics of $H_{x}(x,z,t)$. Minimum value points were selected similar to those in the analysis on $H_{z}(x,z,t)$. Then, the z position and time degree curves were calculated and plotted in Figure 8. Meanwhile, the velocity and time degree curves were calculated and plotted in Figure 9.

Figure 8 shows that the minimum value points on the three side lines vary with time degrees. The three curves possessed a similar tendency. The curves decreased from 0° to 90° and increased from 90° to 180°. This tendency proves that the minimum value points move with time.

**Figure 10:**
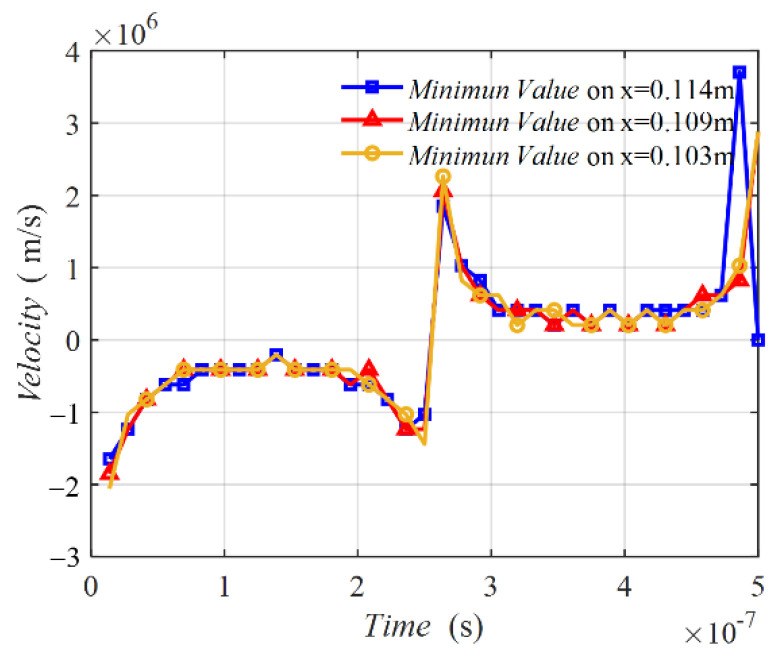
Velocity on three middle lines varying with time.

Figure 9 presents the velocities of the minimum value points, which were not constant. In the first half period of the magnetic field, the velocities were negative and had two peaks, implying that $H_{x}(x,z,t)$ moved from coil 2 to coil 1. In the last half period, the velocities were positive and also had two peaks, implying that $H_{x}(x,z,t)$ moved from coil 1 to coil 2. The velocity curve of $H_{x}(x,z,t)$ was similar to that of $H_{z}(x,z,t)$ shown in Figure 6. Those two motions were the forward and backward waves of the MIW in the WPT system. Figure 10 is the MIW velocity with time, which transferred the results from Figure 9 from time degrees to time. This result shows the velocity in m/s, which presents a better understanding of the MIW velocity.

Table 2 shows the average velocities of $H_{x}(x,z,t)$. The total average velocity was $6.92\;\times \;10^{5}\;m/s$, which was also close to the estimated velocity $8\;\times \;10^{5}\;m/s$ using (17). This result proves the correctness of the estimation Equation (17) and the existence of MIWs in $H_{x}(x,z,t)$.

## 4. Generalization

### 4.1. General WPT Model

An exact velocity equation is difficult to obtain in theory because the magnetic field equations contain T(x,z) functions, as shown in (6) and (7). The velocity is also not a constant based on the velocity analyses shown in Figure 6 and Figure 9.

However, the WPT magnetic field physical process could be simplified into two standing waves, which were separated by distance and had a time phase difference. This simplification was based on the near-field coupling steady state of the WPT system.

First, in the coupling steady state, the coils of a WPT system possess currents, and those currents will not influence each other when the system enters a steady state. Hence, the current in the different coils can be regarded as an independent source.

Second, in the near-field condition, the current source will not radiate waves into the far field. Thus, in the near field of the WPT system, the magnetic field H(x,z,t) is composed of the two magnetic field components $H_{1}(x,z,t)$ and $H_{2}(x,z,t)$, which are generated by different coils. The component on the field point will vary with time variation, which is the variation in current. This result can be found in the following equation:

(18) $$\begin{array}{ll} H(x,z,t) & \;=\;H_{1}(x,z,t)\;+\;H_{2}(x,z,t)\\ & \;=\;H_{x1}(x,z,t)e_{x}\;+\;H_{z1}(x,z,t)e_{z}\\ & \;+\;H_{x2}(x,z,t)e_{x}\;+\;H_{z2}(x,z,t)e_{z}\\ & \;=\;I_{1}\cos(\omega t)T_{x1}(x,z)e_{x}\;+\;I_{1}\cos(\omega t)T_{z1}(x,z)e_{z}\\ & \;+\;I_{2}\cos(\omega t\;+\;∆\phi )T_{x2}(x,z)e_{x}\;+\;I_{2}\cos(\omega t\;+\;∆\phi )T_{z2}(x,z)e_{z} \end{array}$$

Therefore, the simplification model for analyzing the wave motion of a WPT system can be established. The sine wave of the time variation remains, and T(x,z) function is simplified to a distance sine function. Hence, two standing waves can be expressed as

(19) $$A_{1}(z,t)\;=\;\sin z\sin\omega t$$

(20) $$A_{2}(z,t)\;=\;\sin(z\;+\;∆z)\sin(\omega t\;-\;∆p)$$

∆p is the degree difference. ∆z is the distance of the source points of the two standing waves. The composed field B(z,t) is calculated as follows:

(21) $$\begin{array}{ll} B(z,t) & \;=\;A_{1}(z,t)\;+\;A_{2}(z,t)\\ & \;=\;\sin z\sin\omega t\;+\;\sin(z\;+\;∆z)\sin(\omega t\;-\;∆p) \end{array}$$

Equation (21) is a simplified form of (18). It is a composed result of two standing waves, but it contains a traveling wave. This equation can be used to calculate the velocity of the traveling wave.

Let

(22) sinzsinωt + sin(z + ∆z)sin(ωt − ∆p) = 0

and the velocity can be calculated as

(23) $$\begin{array}{l} \cos z\sin\omega t\frac{dz}{dt}\;+\;\omega \sin z\cos\omega t\\ \;+\;\cos(z\;+\;∆z)\sin(\omega t\;-\;∆p)\frac{dz}{dt}\;+\;\omega \sin(z\;+\;∆z)\cos\omega t\;=\;0 \end{array}$$

The result of (23) is

(24) $$\frac{dz}{dt}\;=\;\frac{\;-\;\omega \cos\omega t\left[\sin(z\;+\;∆z)\;+\;\sin z\right]}{\cos z\sin\omega t\;+\;\cos(z\;+\;∆z)\sin(\omega t\;-\;∆p)}$$

Equation (24) implies that the velocity of the traveling wave exists, and it is a function of the frequency, position, and time.

### 4.2. Analysis of the MIW of the General Model

**Figure 11:**
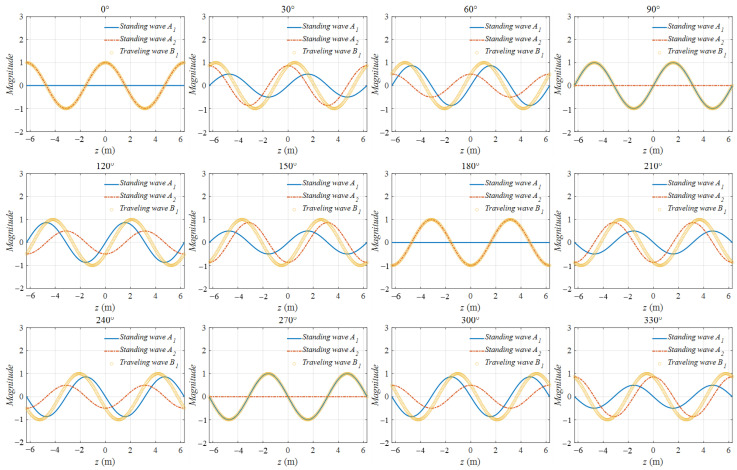
Phenomenon of two standing waves generating a traveling wave.

The velocity shown in (24) is related to ∆z and ∆p. Hence, ∆z is set to 2/π m, and ∆p is set to 90°, 60°, and 30° to present more situations to analyze. The related B(z,t) under three conditions is $B_{1}(z,t)$, $B_{2}(z,t)$, and $B_{3}(z,t)$. The variations in the two standing waves $A_{1}(z,t)$ and $A_{2}(z,t)$ and the traveling wave $B_{1}(z,t)$ are presented in Figure 11.

In Figure 11, the blue line stands for $A_{1}(z,t)$, and the dashed red line stands for $A_{2}(z,t)$. Those two waves were pulsing on different z points and did not possess the wave traveling motion along the z direction when the time degree varied. However, $B_{1}(z,t)$, which is expressed by the circle yellow line, moves along the z direction when the time degree varies from 0° to 180°. The animation is presented on this journal’s website.

**Figure 12:**
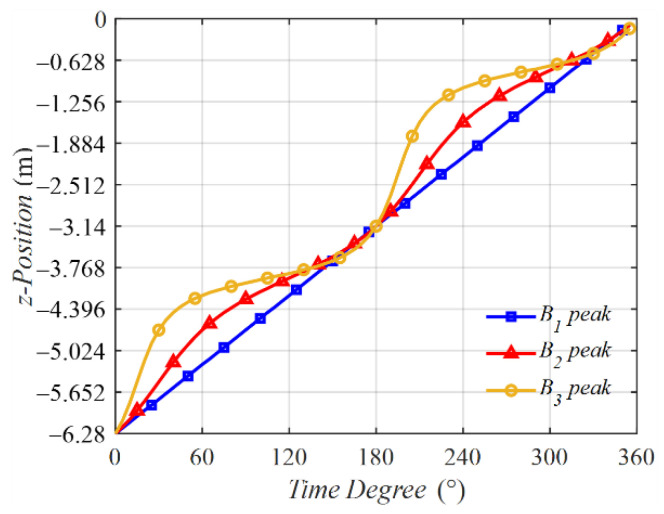
Z positions of the peak points of $B_{1}(z,t)$, $B_{2}(z,t)$, and $B_{3}(z,t)$ varying with the time degrees.

**Figure 13:**
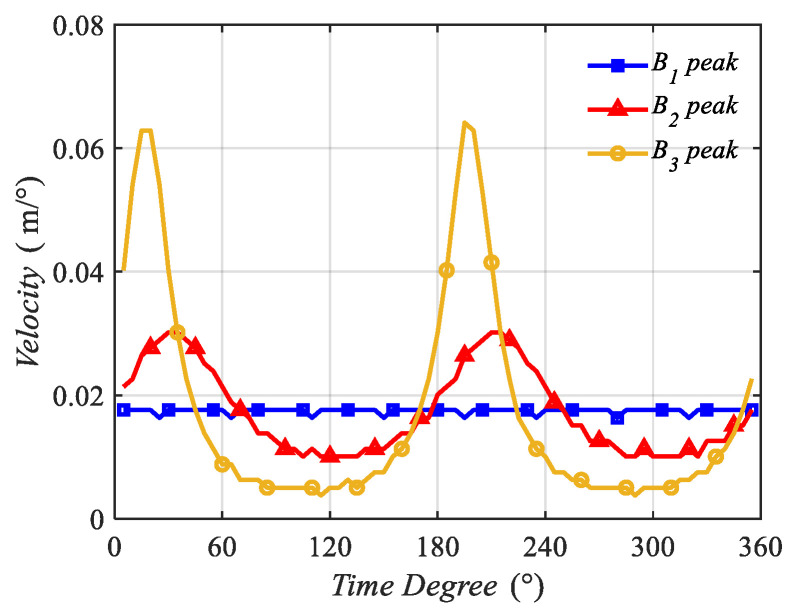
Velocities of the peak points of $B_{1}(z,t)$, $B_{2}(z,t)$, and $B_{3}(z,t)$ varying with the time degrees.

The peak points of $B_{1}(z,t)$, $B_{2}(z,t)$, and $B_{3}(z,t)$ from z = −6.28 m to z = 0 m were selected to calculate the traveling velocity. The z position variations with the time degrees of the peaks of the three traveling waves were calculated and are plotted in Figure 12. The instantaneous velocities of the peak points of $B_{1}(z,t)$, $B_{2}(z,t)$, and $B_{3}(z,t)$ were calculated and are shown in Figure 13.

In Figure 12, the blue line stands for the peak point of $B_{1}(z,t)$, and the blue line is in a linearity, implying that in the condition $∆p\;=\;90^{\circ }$, the velocity of $B_{1}(z,t)$ is a constant. However, this result is a special case. More generally, when $∆p\;\neq \;90^{\circ }$, the instantaneous velocity of $B_{2}(z,t)$ or $B_{3}(z,t)$ is not a constant.

In Figure 13, the instantaneous velocities are plotted, showing that on the condition that $∆p\;\neq \;90^{\circ }$, the velocities are not a constant and possess peaks during a period.

The average velocity was calculated and is shown in Table 3. The estimated average velocity was calculated according to Equation (17), and the result is $\bar{v}\;=\;4fd\;=\;4\frac{\pi }{2}\frac{1}{2\pi }\;=\;1(m/s)$. This result was close to the average velocities calculated using theoretical analysis, which are presented in Table 3, and proves that the estimating equation is correct.

The analysis of the general model not only proves the correctness of the analysis on the wave moving motion characteristics but also reveals a physical phenomenon that two standing waves can generate a traveling wave. These results extend the mechanism of the MIW in a WPT system into a more general situation.

## 5. Experiment

**Figure 14:**
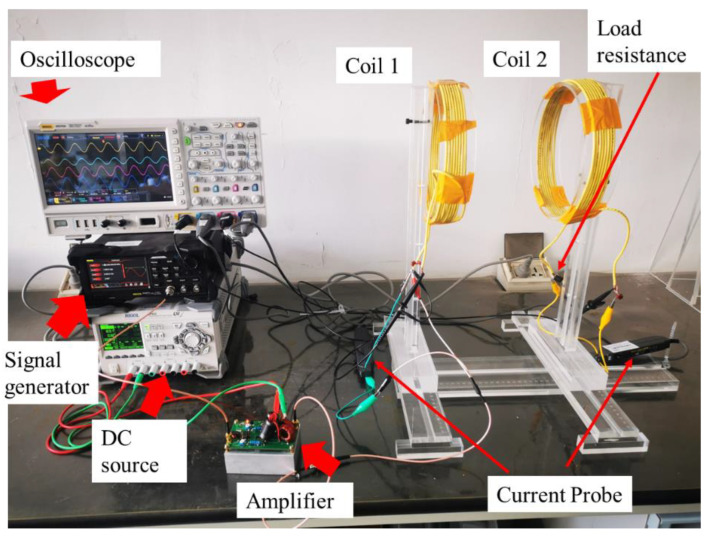
The two-coil WPT system in the experimental stage.

**Figure 15:**
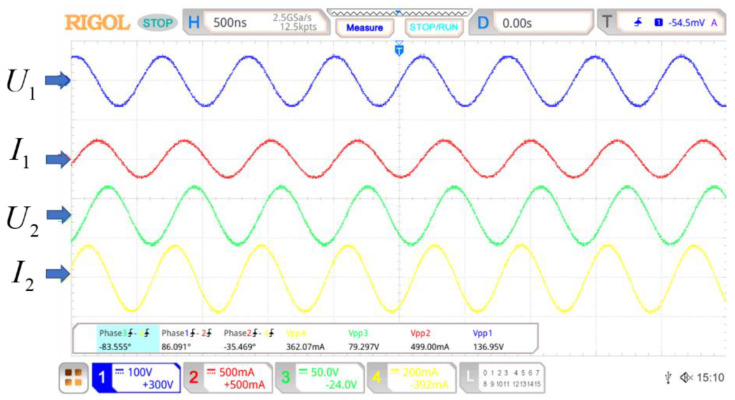
The voltages and currents of the WPT system during the experiment.

A WPT system was set up to show the operation state of the real system, which is shown in Figure 14. Two eight-turn coils were in this system, and the distance of the two coil was 0.2 m. The working frequency was tuned to 1.515 MHz. Both coils were 24 uH. The voltages and currents of the system are shown in Figure 15. U1 = 136.95 V and U2 = 79.30 V stand for the voltages of coils 1 and coil 2, respectively. I1 = 0.499 A and I2 = 0.362 A were the currents in coils 1 and coil 2, respectively. The phase difference of I1 and I2 was −35°. The coil system efficiency was 63%. Under this experiment state, the velocity of the MIW could be calculated according to (17). The velocity of the MIW in this experiment state was $\bar{v}\;=\;4df\;=\;4\;\times \;0.2\;\times \;1.515\;\times \;10^{6}\;=\;\;1.212\;\times \;10^{6}\;m/s$. These results demonstrate that the theoretical analysis can be applied to a real system.

## 6. Verification

A simulation was conducted to verify the correctness of the analysis. The simulation used the ANSYS Electromagnetics Suite. Initially, a WPT model with two coils was built. This model was the same as the model in the analysis shown in Figure 1.

**Figure 16:**
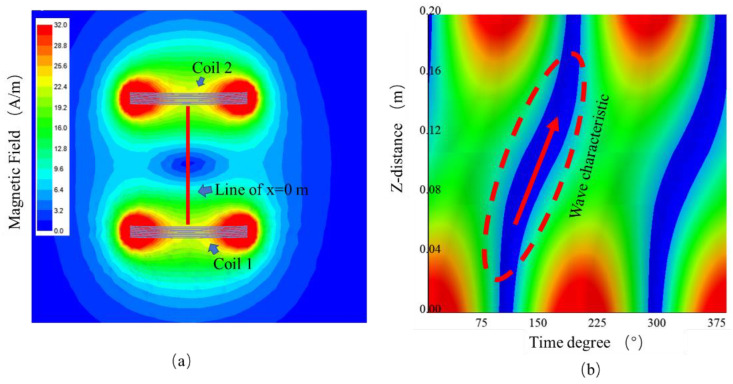
Simulation results: (**a**) magnetic field distribution at the time degree equal to 135°, (**b**) magnetic field on the line of x = 0 varying with time degrees.

The simulation results are shown in Figure 16. Figure 16a shows the magnetic field distribution at the time degree equal to 135°, and a dark blue region appears in the middle of the two coils. The red line is the line of x = 0 m. The magnetic field on this line varies with time degrees, as shown in Figure 16b. In Figure 16b, the *x*-axis is the time degree from 0° to 375°, and the *y*-axis is the z direction from 0 m to 0.2 m, indicating the z direction of x = 0 m line. A blue S curve can be observed; this curve is the wave moving characteristic, which is the MIW. As the time degree increases, the curve rises. This curve represents the minimum value point moving from coil 1 to coil 2. These results prove the existence of the MIW characteristic.

**Figure 17:**
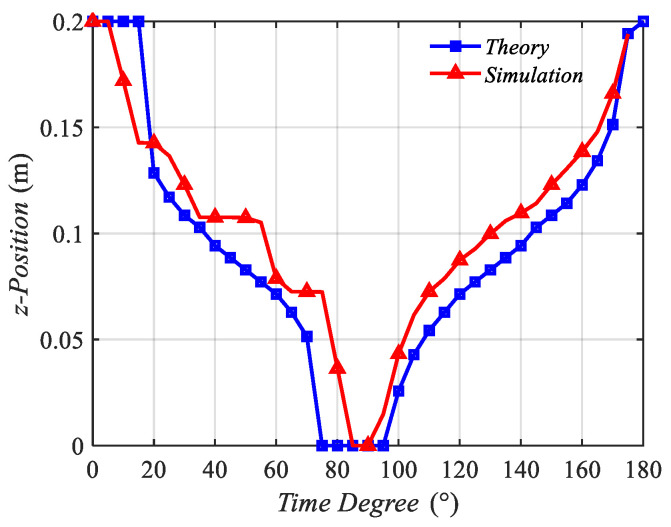
Verification of the theoretical analysis.

The minimum points of the simulation and theoretical analysis are plotted in Figure 17. The two curves in Figure 17 demonstrated the same tendency, proving the correctness of the analysis. The average velocity of the magnetic field was calculated, which is $8.1\;\times \;10^{5}m/s$. This average velocity obtained by the simulation was close to the average velocity calculated by the estimating Equation (17) and the analysis results. These results prove the correctness of the estimating equation and the theoretical analysis.

## 7. Conclusions

This study analyzes the MIW characteristics of the magnetic field in a WPT system. This study presents a novel view of the mechanisms of the WPT system from a wave view. The findings in this study can be used to analyze the mechanisms of the WPT system. This study also expands the knowledge of the MIW in a real system.

First, a wave motion phenomenon was found in the instantaneous distributions of the magnetic field. The wave motion appears in the middle region and side region of the space between coils shown in Figure 1.

Second, the MIW was quantified. Target points of the magnetic field were selected, and the instantaneous velocities were calculated. The velocity curves showed that the velocity of the MIW was not a constant but was a function of the time and space position. The instantaneous velocity of the magnetic field in the WPT system was considerably less than the velocity of the plane wave in free space. This velocity of the magnetic field was a kind of phase velocity. We also found an estimating Equation (17) that could be used to estimate the average velocity of the MIW, and the accuracy of this equation has been proven using analysis.

Third, a simplification model for the magnetic field of a WPT system was built to analyze the MIW characteristics. The results show that two standing waves can generate a traveling wave. This physical phenomenon can be used to illustrate not only the MIW characteristics of the magnetic field in a WPT system, but also that in other systems in a near-field coupling steady state.

Lastly, a verification was conducted, and the correctness of the analysis in this paper has been proven.

This study promotes the analysis of MIW theory from a relay coil system to a general situation and presents a deep insight into the instantaneous characteristics of the magnetic field in WPT systems. In the future, the MIW could be analyzed in more complex situations, such as the coil deviation situation which was not analyzed in this study.

## Figures and Tables

**Table 1:** Average velocities of $H_{z}(x,z,t)$.

Velocity (M/S) On Different Lines	From 0° to 90°	From 90° to 180°
x = 0 m	$8.47\;\times \;10^{5}$	$1.20\;\times \;10^{6}$
x = 0.03 m	$8.47\;\times \;10^{5}$	$1.03\;\times \;10^{6}$
x = 0.06 m	$8.00\;\times \;10^{5}$	$8.47\;\times \;10^{5}$
Average velocity	$8.31\;\times \;10^{5}$	$1.03\;\times \;10^{6}$
Total average velocity	$9.28\;\times \;10^{5}$	

**Table 2:** Average velocities of $H_{x}(x,z,t)$.

Velocity (m/s) On Different Lines	From 0° to 90°	From 90° to 180°
x = 0.114 m	$7.26\;\times \;10^{5}$	$6.86\;\times \;10^{5}$
x = 0.109 m	$6.86\;\times \;10^{5}$	$6.86\;\times \;10^{5}$
x = 0.103 m	$6.86\;\times \;10^{5}$	$6.86\;\times \;10^{5}$
Average velocity	$6.99\;\times \;10^{5}$	$6.86\;\times \;10^{5}$
Total average velocity	$6.92\;\times \;10^{5}$	

**Table 3:** Average velocities in different phase conditions.

	$B_{1}(∆p\;=\;90^{{}^{\circ}})$	$B_{2}(∆p\;=\;60^{{}^{\circ}})$	$B_{3}(∆p\;=\;30^{{}^{\circ}})$
Average velocity (m/s)	1.000	0.998	0.990

## Data Availability

Not applicable.
